# Diagnostic Approach in a Rare Lung Tumor: Inflammatory Myofibroblastic Tumor

**DOI:** 10.1155/crpu/8877404

**Published:** 2025-12-26

**Authors:** Justine Po, Lamia Aljundi, Giselle Kim, Monika Kakol

**Affiliations:** ^1^ Keck School of Medicine, University of Southern California, Los Angeles, California, USA, usc.edu; ^2^ Division of Pulmonary and Critical Care, Keck School of Medicine of University of Southern California, Los Angeles, California, USA, usc.edu

## Abstract

Diagnosis and management of inflammatory myofibroblastic tumors (IMTs) are challenging due to limited data and complications with existing techniques. We report a case of IMT in which a patient was found to have a rare near‐total obstructive endobronchial tumor in the left mainstem bronchus after two unsuccessful bronchoscopy attempts—the second attempt requiring intubation due to significant bleeding, which is a common finding in IMTs. We suggest using cryorecanalization for tumor debulking to minimize the risk of bleeding during bronchoscopies and to follow up with argon plasma coagulation (APC) for safe management. The patient tolerated this procedure well with no complications and had improved symptoms, emphasizing the importance of innovative patient‐tailored care to treat rare and complex lung tumors such as IMTs.

## 1. Introduction

Inflammatory myofibroblastic tumors (IMTs) are rare pathological entities [1] that can pose significant diagnostic challenges because of their uncommon nature and nonspecific and variable presentation [2]. Multiple case reports have also noted IMTs′ propensity to bleed, which may necessitate further workup as the patient′s chief complaint [3, 4] but may also make biopsy challenging [5, 6].

This case report highlights our approach to the challenges faced in diagnosing and managing a patient with an IMT presenting as a near‐total obstruction of the left mainstem bronchus. Central airway obstructions are serious and potentially life‐threatening conditions, requiring the involvement of skilled interventional pulmonologists for effective management. This report also delves into the variable treatment options available for managing IMTs. The rarity of these tumors poses a diagnostic challenge, while controversy over whether IMT represents a neoplastic versus reactive process has led to variable approaches to systemic therapy in the literature [2].

Given the rarity and complexity of these tumors, diagnostic and treatment decisions must be tailored to each patient′s specific clinical scenario. We discuss our strategy and provide insights into the dynamic nature of managing such rare lung tumors.

## 2. Case Presentation

A 54‐year‐old nonsmoker female casino worker with occupational exposure to secondhand smoke reported to her primary care provider with a 2‐year history of progressively worsening dyspnea, orthopnea, cough, and left‐sided chest pain. The patient had no pertinent past medical history, drank 1–2 glasses of wine per week, and denied any history of drug abuse. Initial evaluation led to hospitalization for pneumonia. Although the patient′s respiratory symptoms largely resolved after treatment, a chest computed tomography (CT) scan during her hospitalization revealed an endobronchial mass with near‐total obstruction of the left mainstem bronchus (Figure 1a). A combined PET/CT scan exhibited intense fluorodeoxyglucose (FDG) with a maximum standardized uptake value (SUV_max_) of 29.2 (Figure 1b).

Figure 1(a) CT scan of the chest demonstrating obstruction of left mainstem bronchus. (b) CT/PET scan demonstrating an FDG avid mass centered at the left main bronchus with the maximum SUV = 29.2, a score consistent with malignancy.

**Figure:**
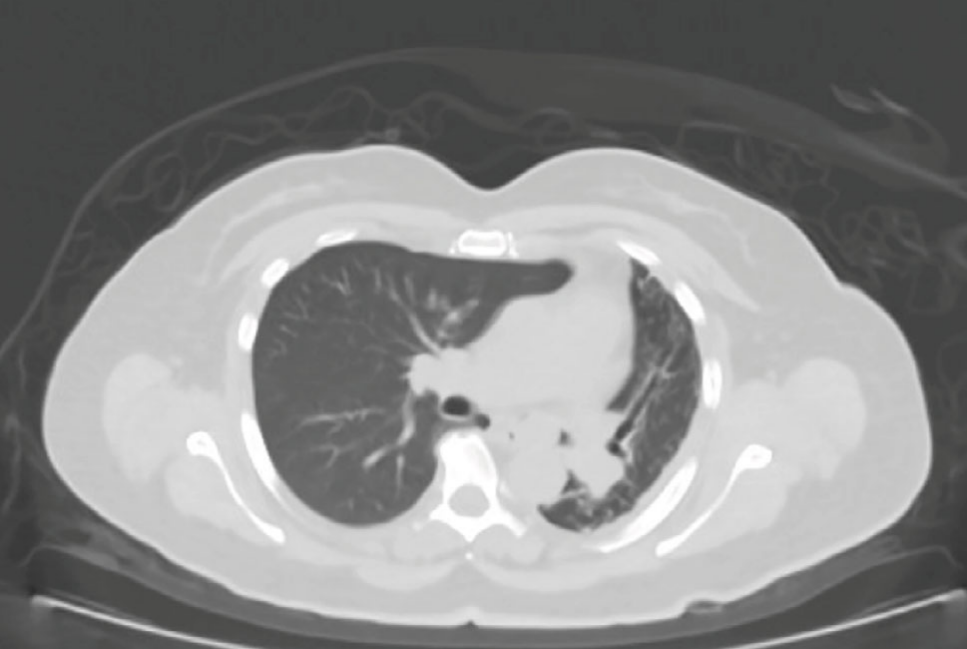
(a)

**Figure:**
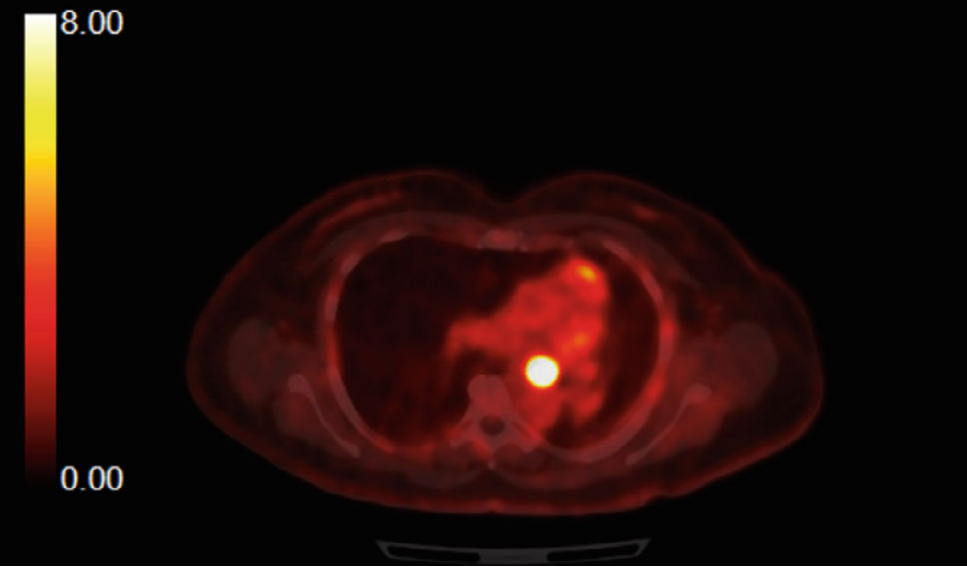
(b)

The patient subsequently underwent an initial flexible bronchoscopy, which was nondiagnostic. A second attempt was performed; however, the procedure was aborted due to significant bleeding that necessitated overnight intubation for airway management. After stabilization and discharge, the patient was referred to our tertiary academic care center for further diagnostic workup, with interventional pulmonary and thoracic surgery consultation. Preoperative assessment revealed that the patient remained hemodynamically stable with no evidence of active bleeding; thus, a contrast‐enhanced CT was not performed. Recognizing the tumor′s highly vascular nature, we employed multiple approaches to minimize bleeding risk during the operation.

### 2.1. Procedure

**Figure 2:**
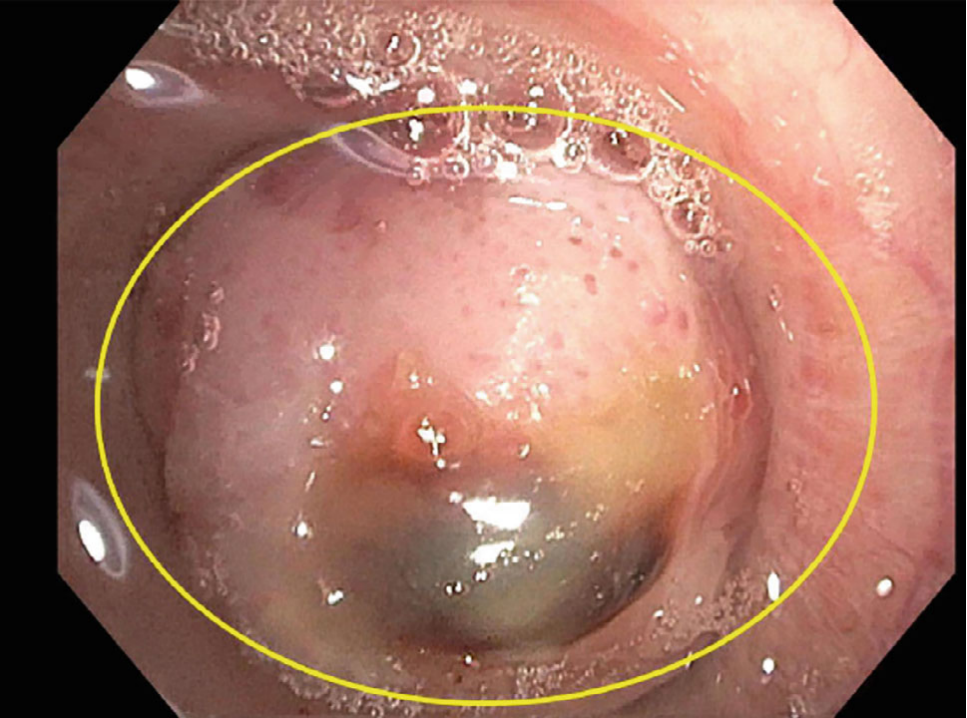
Tumor obstruction of the left mainstem bronchus.

The patient underwent flexible bronchoscopy under general anesthesia. The patient was first intubated with an 8.5 endotracheal tube, after which an Olympus bronchoscope with a 2.8 inner diameter channel was utilized for airway examination. A friable, fungating, and smooth mass was identified within the left mainstem bronchus, estimated to be obstructing greater than 90% of the airway (Figure 2).

After traversing the left endobronchial mass, the bronchoscope was withdrawn and replaced with an endobronchial ultrasound (EBUS) bronchoscope for lymph node examination. Sampling by transbronchial needle aspiration (TBNA) was also performed using an Olympus EBUS‐TBNA 22‐gauge needle in the right lower paratracheal region (Station 4R), subcarinal mediastinum (Station 7), and right superior interlobar region (Station 11Rs). Rapid on‐site evaluation (ROSE) of the specimens was conducted to ascertain adequate cellularity.

Endobronchial biopsies of the obstructing mass were performed in the left mainstem bronchus using a 21‐gauge needle and forceps. Both the endobronchial and lymph node biopsy samples were then sent for cell count, bacterial culture, viral smears and culture, and fungal and acid‐fast bacilli analysis and cytology.

**Figure 3:**
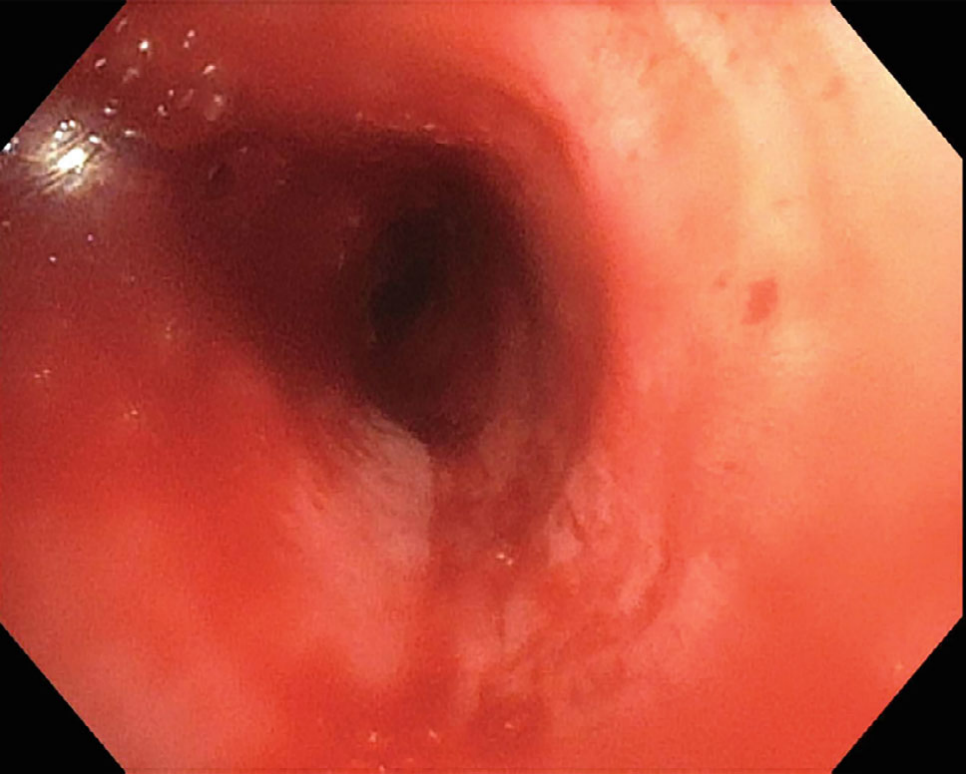
Left mainstem post‐tumor debulking.

Tumor debulking was achieved through an alternating approach of cryotherapy and argon plasma coagulation (APC). Cryotherapy was executed with a 2.9 mm cryoprobe, facilitating the progressive excision of the tumor with a freezing duration of 20 s and a thawing duration of 5 s. APC was employed intermittently at 20 W of pulse energy, effectively stopping bleeding (Figure 3).

### 2.2. Outcomes

Bronchoscopic intervention achieved complete recanalization of the airway. The patient tolerated the procedure well with no complications reported and was discharged the same day. In the follow‐up visit, the patient reported a notable improvement in symptoms.

The pathology report demonstrated the presence of spindle cells arranged in fascicles admixed with CD31‐positive inflammatory cells, predominantly plasma cells. Immunostains showed diffuse positivity for ALK‐1, patchy positivity for desmin, focal positivity for SMA, and negativity for AE1/AE3, HHV8, ERG, and S100. Altogether, these findings were consistent with the diagnosis of IMT. Biopsy of the mediastinal lymph nodes demonstrated normal lymphoid tissue, suggesting localized disease.

The patient′s case was discussed at a multidisciplinary tumor board meeting, and consensus was for surveillance noncontrast chest CT scan in 3 months′ time. The repeat CT scan demonstrated partial recurrence of the tumor in the left mainstem bronchus (Figure 4a).

Figure 4(a) CT chest 3 months after bronchoscopy. (b) Repeat bronchoscopy showing recurrence of endobronchial tumor.

**Figure:**
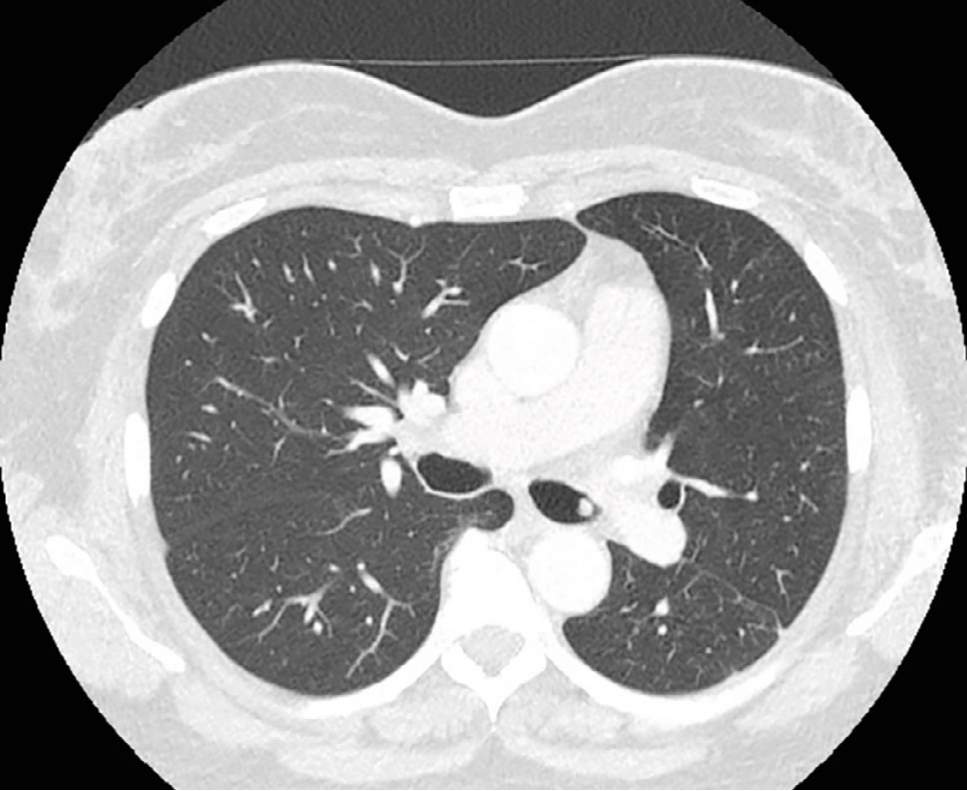
(a)

**Figure:**
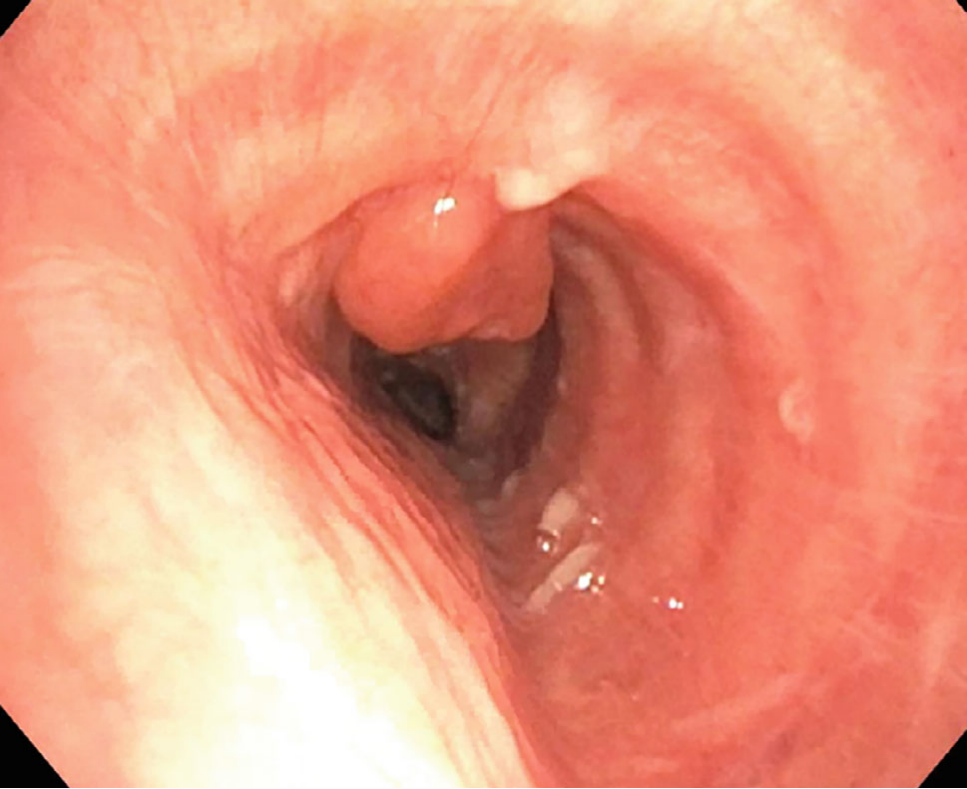
(b)

Clinically, the patient reported her breathing to remain improved since the procedure, though with some recurrence of dyspnea on exertion. The thoracic surgical team requested repeat bronchoscopy, which confirmed a 2.5 × 2.5 mm friable, infiltrative, raised, and smooth nonobstructing mass (Figure 4b).

Biopsies were recollected, with pathology still consistent with IMT and fluorescence in situ hybridization (FISH) analysis showing ALK rearrangement. The patient then underwent thoracotomy with sleeve resection of the left mainstem bronchus and has been stable for the past months.

## 3. Discussion

IMT is defined by the Connective Tissue Oncology Society as an ultrarare sarcoma, with an incidence of less than 1 in 1,000,000 annually [7]. IMTs represent 0.04%–1% of all lung lesions [1] and most often occur in adolescents and young adults [2, 8], though the primary tumor site and patient demographics can vary widely [2].

Among pulmonary IMTs, this patient′s presentation of a near‐total obstructive endobronchial tumor represents a rarity. In a retrospective case series of pulmonary IMTs, only one patient (5.8% of cases) presented with an endobronchial tumor, as seen in this patient, while other case series have reported endobronchial IMTs representing 10%–20% of pulmonary IMT cases [9]. The patient′s high SUV_max_ indicates markedly increased metabolic activity, which is also uncommon among IMTs [10]. A case series correlating FDG‐PET/CT findings with pathology in patients with IMTs found an average FDG uptake of SUV_max_ of 10.9 ± 5.5, with stronger FDG uptake associated with more aggressive pathologic features of higher cellularity, proliferative index, and atypia. In this patient, higher SUV_max_ may have been associated with more aggressive IMT features, contributing to her tumor recurrence [9, 11].

In general, contrast‐enhanced CTs can help evaluate vascular anatomy and potential bleeding sources. However, in this patient, noncontrast CT and PET/CT had already demonstrated a localized, endobronchial mass without intraparenchymal extension or features suggestive of an arteriovenous malformation. Given these imaging findings and the patient′s hemodynamic stability, the minimal expected benefit of an additional contrast‐enhanced study was outweighed by the potential risks of added radiation and contrast exposure. Thus, proceeding directly with bronchoscopy to restore airway patency and obtain a histologic diagnosis was deemed both safe and appropriate in this context.

IMTs may be difficult to diagnose preoperatively [9]. At the same time, thorough preoperative evaluation, including preoperative biopsy and immunohistochemistry, may facilitate better outcomes. Given the heterogeneity of IMTs, such evaluation could help to reduce surgical morbidity and inform consideration for neoadjuvant therapy in patients with large ALK‐positive tumors [12]. This patient experienced two previous failed diagnostic bronchoscopies at outside hospitals, with the second attempt aborted due to significant bleeding. This observation is in line with previous case reports that have noted IMTs′ propensity to bleed [5, 6]. To address this risk during bronchoscopy, a critical aspect of our approach was the use of cryoablation for tumor debulking, utilizing APC for precise coagulation and cryotherapy for tissue devitalization. Our institution serves as a referral center for patients with highly challenging cases, including friable airway tumors following unsuccessful interventions at outside hospitals. We are equipped with immediate access to endobronchial occlusion devices, diluted epinephrine, and tranexamic acid (TXA) for the management of significant intraprocedural bleeding. While these adjunctive measures were available, they were not required in this case. The controlled freezing and thawing cycles allowed for effective reduction of the mass while minimizing the risk of bleeding. Post‐cryotherapy, APC provided a further safeguard to manage residual bleeding effectively. In the setting of this patient′s previous nondiagnostic bronchoscopies, ROSE during needle aspiration proved useful to ensure adequate sampling. A recent study found 23.5% of pulmonary IMTs to exhibit low cellularity [9], supporting the benefit of ROSE to minimize the need for rebiopsy.

IMTs are known for a low potential for recurrence [2]. Though limited by a small sample size, studies have reported local recurrence rates ranging from 0% to 38%, with an 11% rate of metastasis [9, 11, 12]. While not seen in this case, a rare and aggressive subtype of IMT known as epithelioid inflammatory myofibroblastic sarcoma (EIMS) has been previously described, which appears with epithelioid or round cell morphology and expresses different fusion genes (most frequently RANBP2‐ALK fusion) [2, 8].

The overall indolent nature of IMTs, in combination with the readily accessible location of this patient′s tumor site by bronchoscopy, without evidence of local invasion or lymph node involvement, led to a conservative approach to initial management. On partial recurrence, pulmonary sleeve resection was recommended, following the standard practice of surgical resection for focal IMTs [2, 8]. We posit that sleeve resection can be a favorable surgical approach for centrally located focal IMTs in order to minimize loss of healthy tissue.

Endobronchial tumor debulking via cryorecanalization combined with APC offers significant advantages in managing airway obstruction. The use of a cryoprobe facilitates immediate relief of airway obstruction while enabling the acquisition of substantial tissue samples for pathological analysis. This method minimizes complications associated with burn or crush artifacts, which are common pitfalls when using APC and forceps. By preserving the integrity of the tissue, cryorecanalization enhances diagnostic accuracy and therapeutic outcomes.

Although bleeding can occur with the use of cryotherapy for tumor debulking, major bleeding is rare. In a study that evaluated 225 bronchoscopic procedures in which cryorecanalization using cryoprobe was utilized, APC was used in 16.4% of the cases, and minor bleeding was defined as bleeding requiring ice‐cold NaCl or epinephrine solution and occurred in 4% of the cases. Moderate bleeding was defined as bleeding requiring APC or a bronchial blocker and occurred in 18% of the cases. No major bleeding was encountered [10].

Based on findings from a recent study conducted at China Children′s Medical Center, systemic therapy is not indicated in focal cases of IMT that demonstrate negative microscopic margins [8]. In this case, if the patient′s IMT were to progress to Stage 2 disease, our team would consider initiating an adjuvant ALK inhibitor, such as alectinib, for 2 years. This decision would be made in the context of this tumor′s known ALK‐1 positivity and ALK rearrangement [2, 13].

To enhance the management of IMTs and reduce the likelihood of local recurrence, adjuvant radiotherapy and corticosteroids have been employed in certain cases [2, 9, 12]. However, establishing a standard chemotherapy regimen for IMTs remains challenging due to their infrequent occurrence and the limited availability of data. Consequently, systemic treatments are typically reserved for cases of advanced disease. In this context, anthracycline‐based and MTX‐V chemotherapy regimens have demonstrated an overall response rate of approximately 50% [2], but this regimen has been largely supplanted by the use of targeted tyrosine kinase inhibitors [2, 14, 15]. Previous research emphasizes the importance of early gene rearrangement identification for accurate treatment, especially in advanced stages of IMT [2].

## 4. Conclusion

IMTs pose significant diagnostic and therapeutic challenges, as evidenced in this case. The complexity of diagnosing such tumors highlights the need for specialized care and innovative treatment approaches. Our management strategy reflects the tailored and dynamic approach required in such cases. This case underscores the importance of a multidisciplinary approach in the management of rare and complex lung tumors.

## Consent

No written consent has been obtained from the patients, as there is no patient‐identifiable data included in this case report.

## Conflicts of Interest

The authors declare no conflicts of interest.

## Funding

No funding was received for this manuscript.

## Data Availability

The data that support the findings of this study are available upon request from the corresponding author. The data are not publicly available due to privacy or ethical restrictions.
